# First report of the bat fly species *Basilia italica* in Romania

**DOI:** 10.3897/BDJ.9.e57680

**Published:** 2021-01-18

**Authors:** Áron Péter, Andrei Daniel Mihalca, Attila D Sándor

**Affiliations:** 1 University of Agricultural Sciences and Veterinary Medicine, Cluj-Napoca, Romania University of Agricultural Sciences and Veterinary Medicine Cluj-Napoca Romania; 2 University of Veterinary Medicine, Budapest, Hungary University of Veterinary Medicine Budapest Hungary

**Keywords:** Chiroptera, distribution, host-parasite relationships, *Myotis mystacinus*, Nycteribiidae

## Abstract

Bat flies are haematophagous ectoparasites, highly specialised to bats and are also considered to have vectorial potential for several pathogens like *Bartonella* spp. or *Polychromophilus* spp. In Romania, past studies mostly focused on the ectoparasitic fauna of cave-dwelling bats, listing the occurrence of 10 bat fly species in the country, with only scarce information on bat flies infesting crevice-roosting bat species. Here we report the occurrence of *Basilia italica*, a rare nycteribiid species infesting primarily forest-dwelling bats. This is the first country-record for Romania and also represents the easternmost occurrence of this species. Further studies are needed to evaluate the vectorial potential of *B. italica*.

## Introduction

Bat flies (Diptera, Hippoboscoidea) are highly specialised ectoparasites of bats representing two families in the superfamily Hippoboscoidea (Streblidae and Nycteribiidae; Reeves and Lloyd 2018). Species belonging to Nycteribiidae are wingless, while most streblids have functional wings (with some exceptions). These dipterans are exclusive parasites of bats and are highly adapted to a parasitic life style. Bat flies are haematophagous and have highly specialised reproductive organs. These parasites reproduce by viviparous puparity and only females leave the host, when larvideposit on the roost-substrate (Dick and Patterson 2006). Most bat flies show high host-specificity (monoxenous), being exclusive parasites of one or a few morphologically similar and phylogenetically closely related bat species (Dick 2007). Bat flies are increasingly recognised as vectors for several pathogen groups like bacteria of the genus *Bartonella* spp. or parasitic protozoa, like *Polychromophilus* spp. (Megali et al. 2010, Dick and Dittmar 2014, Sándor et al. 2018). Although the research interest is growing worldwide, most papers focus on parasites of cave-roosting bats in Romania, listing 10 species (Burghele-Balacescu 1966), namely: *Basilia nana*, *B. nattereri*, *Nycteribia latreillii*, *N. kolenatii*, *N. pedicularia*, *N. schmidlii*, *N. vexata*, *Penicillidia duforii*, *P. conspicua* and *Phthiridium biarticulatum*). Gheorghiu (2006) reported the presence of several species at the Piatra Craiului forest and Sándor et al. (2018) described several new parasite-host species associations, while focusing on the epidemiological aspects of nycteribiid-infestation. Information on the biology, ecology or distribution of bat flies is still scarce to support further conclusions about their epidemiological risk contribution. Most of all, we lack this information on bat flies infesting crevice-roosting bat species. Recent studies of bat-related parasites in Romania provided new information for a checklists of bat tics (Sándor et al. 2019) and their vectorial importance (Hornok et al. 2019, McKee et al. 2019), which also led to the study of bat flies (Sándor et al. 2018). Here we report the first records of a rare bat fly (*Basilia italica*) in Romania, these being the easternmost reports of this species in Europe.

## Material and methods

(Methods used for this study were published at protocols.io with the following DOI: dx.doi.org/10.17504/protocols.io.bi2ykgfw).

**Figure 1.**
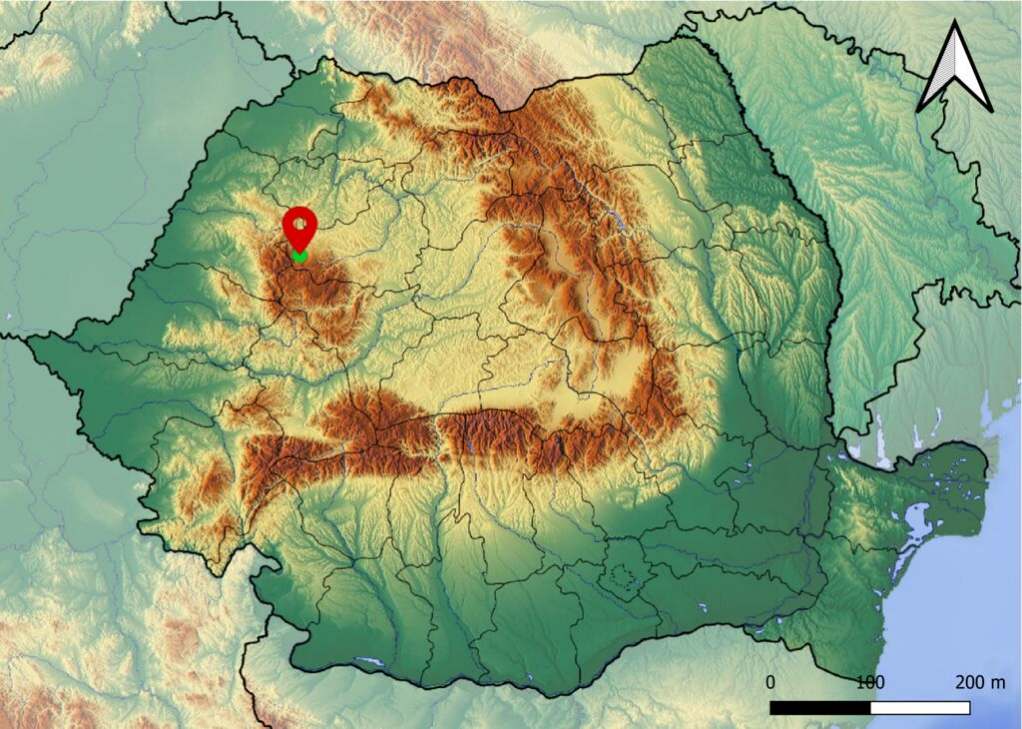
Location of the newly-reported bat fly species *B. italica*.

Bats were trapped using mist nets erected close to the roosting site of an all-male colony of multiple species in an abandoned building close to Ic Ponor (Munții Apuseni, 46.629842N, 22.806450E, Fig. 1), at an altitude of 1044 m on 17.06.2020 and 01.07.2020. Upon capture, bats were examined, species were identified, based on morphological characters (Dietz et al. 2009) and all external parasites were collected using tweezers and were stored in 70% ethanol (single tube/host). In the laboratory, bat flies were identified under stereomicroscope, using morphological keys (Theodor 1967).

## Results

**Figure 2.**
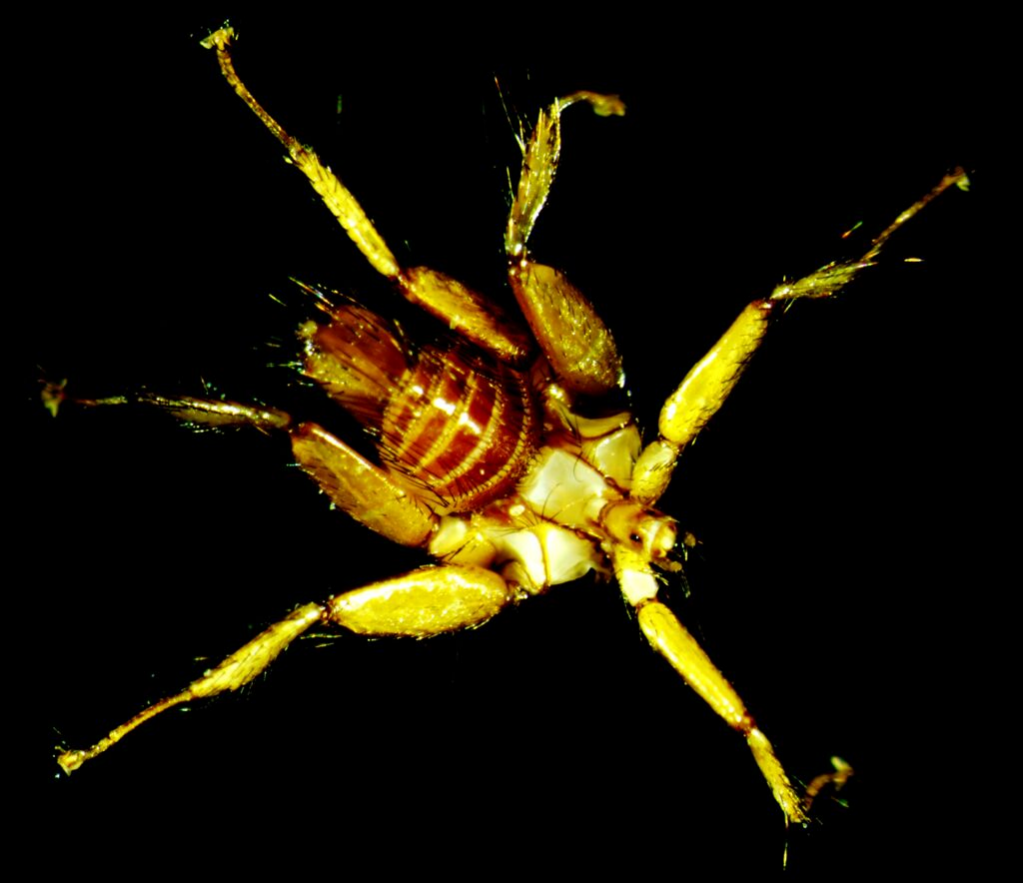
Dorsal view of the male *Basilia italica* caught on *Myotis mystacinus* in Ic Ponor, Munții Apuseni, Romania.

On both trapping occasions, a single bat fly individual was collected from a single adult male *Myotis mystacinus*. None of the other examined individuals (all males, *Vespertilio murinus* – 106, *Pipistrellus pipistrellus* – 17, *M. mystacinus* – 16 and *M. brandtii* - 2) hosted bat flies (Suppl. material 1). Bat flies were identified as a male and a female *Basilia italica*, based on the following morphological characteristics: a small-sized fly with visible eyes, but a body shape similar to the genus of Nycteribiae, a group of spines at the end of sternite 5. The surface of tergites are more or less bare, but on the marginal ends, there are some longer hairs. Lastly, there are long setae on the tibiae and the overall form of sternite 5 is unique (Fig. 2; see Theodor 1967, page 202).

## Discussion

The main host of *B. italica* is *M. mystacinus*, with occasional records on *M. alcathoe* and *M. brandtii* (Aellen 1963, Hůrka 1964, Beaucournu and Noblet 1966, Czuppon and Molnár 2001, Krištofík and Danko 2012, Szentivanyi et al. 2016). In addition, five more bat species were listed as hosts, each with a single record (*Barbastella barbastellus*, *Eptesicus serotinus*, *M. nattereri* - Czuppon and Molnár 2001, *M. emarginatus*, *M. myotis* – Theodor 1967). It is a rare species, with a handful of observations, with a mainly Central European distribution (Hůrka and Soós 1986).

**Figure 3.**
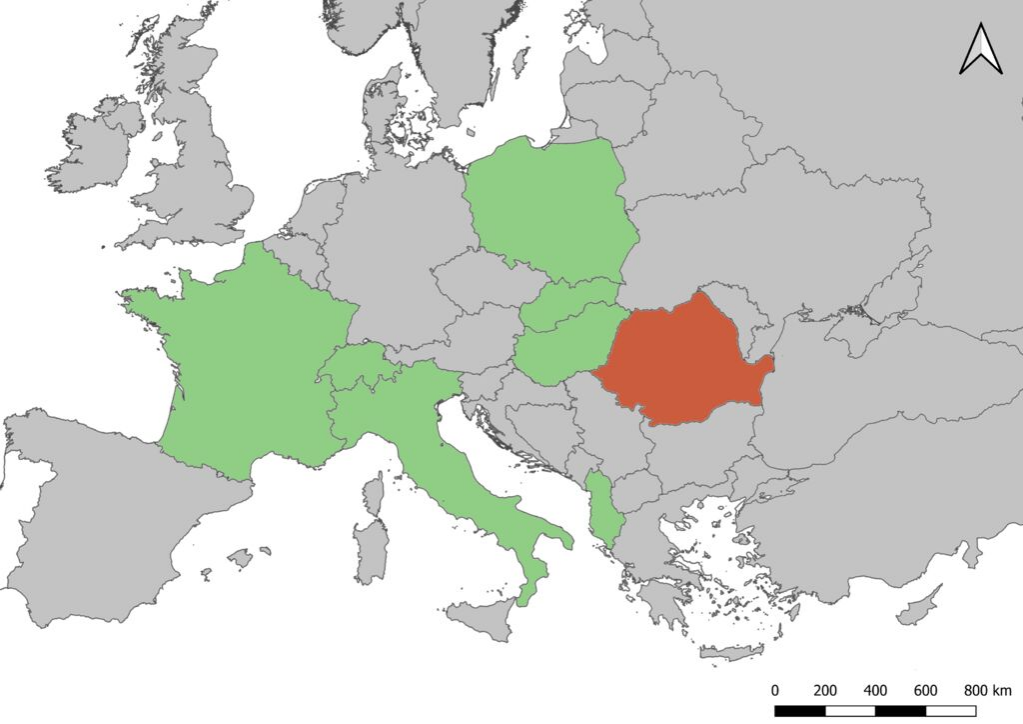
Current geographical distribution of *B. italica* in Europe. Green coloured countries with data from literature by Szentivanyi et al. 2016 and our first record for Romania with red colour.

The finds reported here are the first records of *B. italica* in Romania, hence increasing to 11 the number of known bat fly species in this country. This is a novel and a geographically-distant record, therefore considerably extending the range towards the East. (Fig. 3). The geographically closest occurrence of this fly species is in Hungary, where it was reported from six different species (Czuppon and Molnár 2001, Haelewaters et al. 2017).

Although bat flies are usually abundant on cave dwelling species (like *Miniopterus schreibersii* or *M. myotis*) and their flies are relatively well-known (Zahn and Rupp 2004);, however, the host species of *B. italica* tend to roost in crevices (primarily tree-holes, but also abandoned buildings) and they are captured in smaller numbers (Dietz et al. 2009). After examining almost 500 individuals from the potential host species of *B. italica* in the past five years, these are the first observed individuals of this species in Romania.

## Supplementary Material

AC5F40DD-8147-5639-87BF-17C2B68A5C56 10.3897/BDJ.9.e57680.suppl1 Supplementary material 1 firsOccurenceOfBitalicaInRO_DwC Data type species occurence in DwC Brief description Collection data of the bat fly *B. italica* for the first time in Romania File: oo_438873.xlsx https://binary.pensoft.net/file/438873 Áron Péter, Sándor D. Attila

## Hosting institution

University of Agricultural Sciences and Veterinary Medicine, Cluj-Napoca, Romania
